# Establishment and comprehensive characterization of a subline with highly bone-metastatic propensity derived from the lung adenocarcinoma A549 cell line

**DOI:** 10.1016/j.jbo.2025.100719

**Published:** 2025-10-22

**Authors:** Yujian Xu, Yahan Qin, Wenjun Chai, Ke Xue, Xiaoli Liu, Jing Li, Yue Cao, Lei Sun, Hongyu Pan, Mingxia Yan

**Affiliations:** a Cancer Institute, Fudan University Shanghai Cancer Center, Shanghai 200032, China; b Department of Oncology, Shanghai Medical College, Fudan University, Shanghai 200032, China

**Keywords:** Lung Adenocarcinoma, Bone Metastasis, Cell Model, Tumor Microenviroment

## Abstract

Bone metastasis is a major cause of mortality in lung adenocarcinoma, but the mechanisms remain poorly understood. Here, we established a highly bone-metastatic subline, A549-BM5, from the A549 cell line through five rounds of *in vivo* selection. A549-BM5 cells exhibited enhanced migration and invasion, and preferentially colonized specific skeletal sites in mouse models. In the bone microenvironment, they promoted the recruitment and differentiation of osteoblasts and osteoclasts, disrupting bone homeostasis. Transcriptomic and proteomic profiling revealed dysregulation in pathways such as EMT, adhesion, and bone morphogenesis. We further applied a random forest model to identify potential therapeutic targets associated with bone metastasis. Compared to existing A549-based models, A549-BM5 offers earlier metastasis onset, stable bone tropism, and broader interaction with the bone niche. This model provides a valuable platform for mechanistic studies and therapeutic development targeting lung cancer bone metastasis.

## Highlights

• A549-BM5, a bone-metastatic subline of A549, was established through in vivo selection.
• The model facilitates investigation of mechanisms underlying NSCLC bone metastasis.
• Findings may assist in developing strategies to improve outcomes for lung cancer patients.

## Graphical abstract

**Figure:**
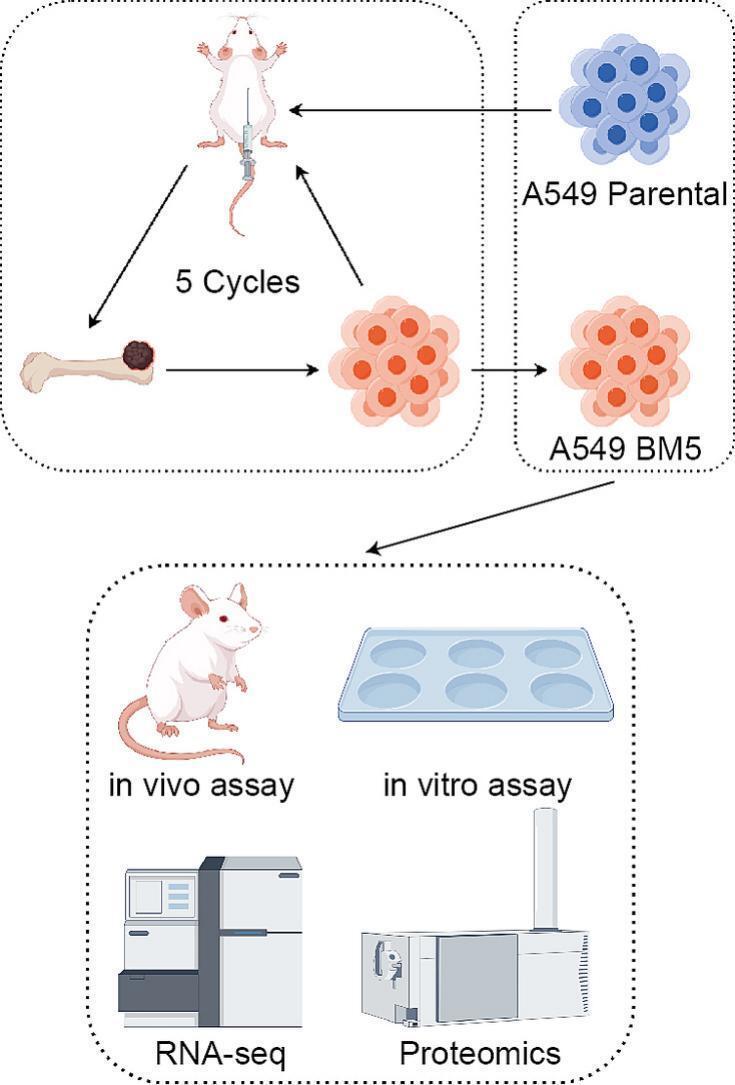

## 1 Introduction

Lung cancer remains a leading cause of cancer-related mortality worldwide [1], with bone metastases representing one of the most common and devastating complications in non-small cell lung cancer (NSCLC). At the time of diagnosis, 20–30 % of NSCLC patients already exhibit bone involvement, and during disease progression, 35–60 % will develop bone metastases [[2], [3], [4]]. These skeletal lesions frequently result in severe skeletal-related events (SREs), including pain, pathological fractures, spinal cord compression, and hypercalcemia, all of which markedly diminish patients' quality of life and worsen clinical outcomes [5]. Although bone-targeted therapies such as denosumab have shown benefits in reducing skeletal complications [6], their efficacy remains limited and is often accompanied by adverse events [7]. More importantly, the molecular mechanisms that govern skeletal colonization in lung adenocarcinoma remain poorly understood. A major limiting factor is the lack of lung adenocarcinoma cell lines with intrinsic bone-tropic potential, which restricts both mechanistic insights and the development of clinically relevant preclinical models.

Disseminated tumor cells (DTCs) reaching bone tissue must undergo complex interactions with various bone microenvironment cells at different stages, including the perivascular niche, the osteogenic niche, and the formation of the vicious cycle[8]. Given the complexity of these interactions, selecting appropriate modeling strategies and suitable cell lines is a critical foundation for advancing research on bone metastasis. Among current strategies, methods such as left ventricular injection [9], intra-tibial or femur injection [10], intra-iliac artery injection [11], and tail artery injection [12] have provided powerful approaches for quantitatively investigating various stages of the bone metastasis cascade. However, these methods often suffer from high experimental variability—an issue particularly pronounced in lung cancer bone metastasis research due to the lack of cell lines with bone-tropic potential, leading to high rates of non-bone metastases and inconsistent tumor engraftment. These limitations underscore the continued need for lung cancer models that are not only stable and reproducible, but also faithfully recapitulate the biological features of bone metastasis. Although a few bone-metastatic lung cancer cell lines have been developed, their number remains limited, and each exhibits specific shortcomings in stability, bone tropism, or biological relevance—issues that constrain their broader applicability. This study aims to address these challenges by establishing a more reliable model.

In the present study, we established a highly bone-metastatic subline, A549-BM5, derived from the lung adenocarcinoma A549 cell line through an *in vivo* selection strategy. We systematically characterized its biological behavior both *in vitro* and *in vivo*, demonstrating enhanced bone-homing ability and metastatic tropism. Furthermore, integrative transcriptomic and proteomic analyses revealed key molecular alterations associated with skeletal colonization. This subline offers a robust and biologically relevant platform for dissecting the mechanisms of lung cancer bone metastasis and for evaluating targeted therapies in a preclinical setting.

## 2 Materials and methods

### 2.1 Cell culture

Human lung adenocarcinoma cell line A549 was obtained from the American Type Culture Collection (ATCC). The A549 cells and their derived subline cells were cultured using Ham’s F-12 K medium (Basal Media, L450KJ). All media were supplemented with 10 % fetal bovine serum (FBS) (YeaSen, 40130ES76) and 1 % penicillin–streptomycin solution (Basal Media, S110jv). Cells were incubated at 37 °C in a humidified atmosphere containing 5 % CO2.

### 2.2 *In vivo* tumor metastasis experiments

Female NOD/SCID or BALB/c nude mice aged 6 to 8 weeks were housed in standard specific pathogen-free (SPF) laboratory animal facilities. In the left ventricular injection experiments, 1 × 10^6^ A549-Parental or A549-BM5 cells were injected into the left ventricle under ultrasound guidance. *In vivo* bioluminescent imaging (BLI) was used to monitor tumor burden. After 4 to 5 weeks, the mice were humanely euthanized, and bone tissues were collected for subsequent experiments.

In the intra-iliac artery injection experiments, following previously reported methods [13], briefly, 0.5 × 10^6^ A549-Parental or A549-BM5 cells were injected into the iliac artery of female BALB/c nude mice aged 6 to 8 weeks. At the end of the eighth day, the mice were humanely euthanized, and bone tissues were collected for subsequent experiments.

Micro-CT analysis of excised femora and tibiae was performed. Raw projection images were reconstructed into cross-sectional slices using **NRecon** (Bruker). Quantitative bone morphometric parameters, including bone integrity, bone volume fraction (BV/TV), trabecular thickness (Tb.Th), trabecular number (Tb.N), and trabecular spacing (Tb.Sp), were calculated using **CTAn software** (Bruker). Three-dimensional reconstructions of bone microarchitecture and osteolytic lesions were generated with **CTvox software** (Bruker). All analyses followed ASBMR guidelines.

### 2.3 Cell survival assays

For the cell viability assay, 1 × 10^3^ cells per well were seeded into 96-well plates (Corning, 3599) with three replicate wells per group and incubated overnight to allow for cell attachment. The following day, cell viability was assessed using the CCK8 reagent kit (YeaSen, 40203ES80) according to the manufacturer’s instructions.

For the colony formation assay, 6 × 10^2^ cells per well were seeded into 6-well plates (Corning, 3516) and cultured for 10 to 14 days to enable colony development. Surviving colonies were fixed with methanol for 30 min and subsequently stained with 0.1 % crystal violet solution (Beyotime, C0121) for another 30 min. After thorough washing and air-drying, the number of colonies was counted.

### 2.4 Cell cycle analysis

Cells were collected from 6-cm cell culture dishes, washed twice with phosphate-buffered saline (PBS), and then fixed in 70 % ethanol at − 20 °C overnight. Following centrifugation and removal of the supernatant, the cells were washed once with PBS and stained with 500 µL of propidium iodide (PI) staining solution (BD Biosciences, 550825) for 10 min. The DNA content was analyzed using a flow cytometer (CytoFLEX S; Beckman Coulter).

### 2.5 Migration and invasion assays

Migration and invasion assays were performed using 8 μm pore chambers (Corning, 353097). For the migration assay, 5 × 10^4^ cells in 200 µL of serum-free culture medium were seeded into the upper chamber. For the invasion assay, the upper chamber membranes were pre-coated with Matrigel (Corning, 356234), and 1 × 10^5^ cells in 200 µL of serum-free culture medium were seeded into the upper chamber. In both assays, culture medium containing 10 % fetal bovine serum (FBS) was added to the lower chamber. After incubation for 16–20 h, the cells that had migrated or invaded through the membrane were fixed with methanol for 30 min and stained with 0.1 % crystal violet for 15–20 min. Nine random fields were photographed under a microscope (Olympus, IX73) at a magnification of 100×.

### 2.6 Protein extraction and Western blotting

Cells were washed with prechilled PBS triplicately, then lysed in buffer (Thermo, 78510) supplemented with protease inhibitors (YeaSen, 20123ES50) and phosphatase inhibitors (YeaSen, 20109ES20). Cells were scraped and thoroughly lysed on ice for 30 min. Lysates were centrifuged at 12,000 rpm for 15–20 min, and the supernatants were collected. Protein concentrations were measured using a BCA assay kit (Thermo Fisher Scientific, 23227), and samples were normalized to equivalent concentrations. Following the addition of 5× protein loading buffer (Yeasen, cat. no. 20315ES20), samples were denatured at 100 °C for 10 min and stored at −80 °C. Proteins were then subjected to sodium dodecyl sulfate–polyacrylamide gel electrophoresis (SDS–PAGE) and transferred onto polyvinylidene fluoride (PVDF) membranes (Bio-Rad, 1620177). Membranes were blocked at room temperature for 2 h prior to incubation with primary antibodies. The primary antibodies used in this study were as follows: anti-N-Cadherin(13116, CST), anti-E-Cadherin(3195, CST), anti-ZO-1(8193, CST), anti-ZEB1(3396, CST), anti-Vimentin(5741, CST), anti-Claudin-1(13255, CST), anti-β-Catenin(8480, CST), anti-MMP2(RM8377, Biodragon), anti-MMP3(RM8188, Biodragon), anti-MMP9(RM3763, Biodragon), anti-MMP13(RM8226, Biodragon), anti-CCL3(RM2987, Biodragon), anti-BMP2(BD-PT5651, Biodragon), anti-BMP3(BD-PT0499, Biodragon), anti-BMP6(BD-PT5655, Biodragon), anti-BMP7(BD-PT0503, Biodragon), anti-OPG(ab183910, Abcam), anti-RANKL(BD-PT5404, Biodragon), anti-OPN(RM4342, Biodragon), anti-COL1A1(RM0325, Biodragon), anti-ICAM-1(BD-PT2269, Biodragon), anti-RANK(BD-PT5881, Biodragon), anti-SP7(BD-PN0332, Biodragon),anti-Beta-Actin(HRP-66009, Proteintech) and anti-Vinculin(26520-1-AP, Proteintech). Bands were visualized by enhanced chemiluminescence (Share-Bio, sb-wb011), and densitometry measurements of the bands were acquired with Quantity One software (Bio-Rad).

### 2.7 Isolation of BMSCs and BMMs

Bone marrow mesenchymal stromal cells (BMSCs) and bone marrow–derived monocytes/macrophages (BMMs) were isolated from 6 to 8-week-old C57BL/6J mice. Under sterile conditions, femurs and tibias were dissected, and the ends of the bones were cut. Bone marrow cells were collected by centrifugation at 10,000×*g* for 5 s, and the pellet was subjected to red blood cell lysis for 5 min on ice. Cells were then centrifuged at 450 × g for 3 min, washed once with PBS, and resuspended in α-MEM complete medium. The suspension was plated in 10-cm dishes, and after 24 h, adherent cells were predominantly BMSCs, while non-adherent cells in the supernatant contained mainly monocytes.

### 2.8 Bone microenvironment cell recruitment assay

Bone microenvironment cell recruitment assays were performed using 8 μm pore Transwell chambers (Corning, 353097). A549-Parental and A549-BM5 cells were seeded at appropriate densities in 24-well plates. Bone microenvironment cells (5 × 10^4^ cells in 200 µL of serum-free culture medium) were seeded into the upper chamber. After incubation for 16–20 h, the cells that migrated through the membrane were fixed with methanol for 30 min and stained with 0.1 % crystal violet for 15–20 min. Nine random fields per chamber were photographed under a microscope (Olympus, IX73) at 100× magnification. Statistical analysis was performed using GraphPad Prism 9.

### 2.9 Osteogenic differentiation assay

Under sterile and light-protected conditions, osteogenic induction medium was prepared by mixing 45 mL α-MEM medium, 5 mL fetal bovine serum, 500 µL penicillin–streptomycin solution, 50 µL of 50 mg/mL vitamin C solution, 5 µL of 1 mM dexamethasone solution, and 500 µL of 1 M β-glycerophosphate solution. The medium was stored at 4 °C for future use. Bone marrow mesenchymal stromal cells (BMSCs) or MC3T3-E1 subclone 14 cells were cultured to sufficient quantities and digested with trypsin. Cells were seeded at a density of 5 × 104 cells per well in 24-well plates. After cell adhesion, the medium was replaced with osteogenic induction medium, with medium changes every other day under light-protected conditions. According to the experimental design, conditioned medium (CM) derived from A549-Parental or A549-BM5 cells was mixed at a 1:1 ratio with osteogenic induction medium (final concentration 50 % CM) during the culture period. After 14 days of induction, Alkaline Phosphatase (ALP) staining was performed.

### 2.10 Osteoclast differentiation assay

Osteoclast differentiation induction medium was prepared by adding cytokines RANKL (50 ng/mL) and M−CSF (10 ng/mL) to α-MEM complete medium. Bone marrow macrophages (BMMs) were cultured to sufficient quantities. Adherent BMMs were digested with Versene dissociation solution and seeded into 96-well plates at a density of 1 × 10^4^ cells per well. After cell adhesion, the medium was replaced with osteoclast differentiation induction medium to induce differentiation, with medium changes every other day. According to the experimental design, conditioned medium (CM) derived from A549-Parental or A549-BM5 cells was mixed at a 1:1 ratio with osteoclast differentiation medium (final concentration 50 % CM) during the culture period.

### 2.11 Osteoblast adhesion assay

MC3T3-E1 subclone 14 cells were seeded at an appropriate density in 6-well plates. After the cells adhered, 200,000 cells in 1000 μL suspension were added to each well and incubated at 37 °C for 30 min. The supernatant was discarded, and the wells were washed with phosphate-buffered saline (PBS). Cell images were captured using a fluorescence microscope at 100 × magnification. Five random fields were selected per well. The number of cells was counted, and the average cell number for each sample was calculated. All experiments were performed in triplicate.

### 2.12 RNA isolation and library preparation

Total RNA was extracted using the TRIzol reagent (Invitrogen, CA, USA) according to the manufacturer’s protocol. RNA purity and quantification were evaluated using the NanoDrop 2000 spectrophotometer (Thermo Scientific, USA). RNA integrity was assessed using the Agilent 2100 Bioanalyzer (Agilent Technologies, Santa Clara, CA, USA). Then the libraries were constructed using VAHTS Universal V6 RNA-seq Library Prep Kit according to the manufacturer’s instructions. The transcriptome sequencing and analysis were conducted by OE Biotech Co., Ltd. (Shanghai, China).

### 2.13 RNA sequencing and differentially expressed genes analysis

The libraries were sequenced on an llumina Novaseq 6000 platform and 150 bp paired-end reads were generated. About 50 M raw reads for each sample were generated. Raw reads of fastq format were firstly processed using fastp [14] and the low quality reads were removed to obtain the clean reads. Then about 48 M clean reads for each sample were retained for subsequent analyses. The clean reads were mapped to the reference genome using HISAT2 [15]. FPKM [16] of each gene was calculated and the read counts of each gene were obtained by HTSeq-count [17]. PCA analysis were performed using R (v 3.2.0) to evaluate the biological duplication of samples. Differential expression analysis was performed using the DESeq2 [18]. Q value <0.05 and foldchange >2 or foldchange <0.5 was set as the threshold for significantly differential expression gene (DEGs). Hierarchical cluster analysis of DEGs was performed using R (v 3.2.0) to demonstrate the expression pattern of genes in different groups and samples. The radar map of top 30 genes was drew to show the expression of up-regulated or down-regulated DEGs using R packet ggradar. Based on the hypergeometric distribution, GO [19], KEGG [20] pathway, Reactome and WikiPathways enrichment analysis of DEGs were performed to screen the significant enriched term using R (v 3.2.0), respectively. R (v 3.2.0) was used to draw the column diagram, the chord diagram and bubble diagram of the significant enrichment term. Gene Set Enrichment Analysis (GSEA) was performed using GSEA software [21,22]. The analysis was used a predefined gene set, and the genes were ranked according to the degree of differential expression in the two types of samples. Then it is tested whether the predefined gene set was enriched at the top or bottom of the ranking list.

### 2.14 4D label-free quantitative proteome analysis

#### 2.14.1 Liquid chromatography

Nanoflow reversed-phase chromatography was performed on an EASY-nLC 1200 system (Thermo Fisher Scientific). Peptides were separated in 90 min at a flow rate of 300 nL/min on a 25 cm × 75 μm column (1.6 μm C18, ionopticks). Mobile phases A and B were 0.1 vol% formic acid solution and 80:20:0.1 vol% ACN: water: formic acid, respectively. The total run was 90 min (0 ∼ 66 min, 3–27 % B；66 ∼ 73 min, 27–46 % B；73 ∼ 84 min, 46–100 % B；84 ∼ 90 min, 100 % B) or 60 min (0 ∼ 45 min, 5–27 % B；45 ∼ 50 min, 27–46 % B；50 ∼ 55 min, 46–100 % B；55 ∼ 60 min, 100 % B).

#### 2.14.2 LC-MS/MS analysis

Liquid chromatography was coupled online to a hybrid TIMS quadrupole TOF mass spectrometer (Bruker timsTOF Pro) *via* a CaptiveSpray nano-electrospray ion source. Capillary voltage was 1.5 kV, dry gas temperature was 180 °C, and dry gas flow rate was 3.0 L/min. The dual TIMS analyzer was operated at a fixed duty cycle close to 100 % using equal accumulation and ramp times of 100 ms. We performed DDA in PASEF mode with 10 PASEF scans per topN acquisition cycle. The full MS scan range was set from 100 to 1700 *m*/*z*. The ion mobility range was 0.75–1.4 vs/cm2, and the collision energy range was 20–59 ev.

### 2.15 Database search

The LC-MS/MS raw data were imported in Maxquant (Version1.6.17.0) for labeling free quantification analysis and the search engine was Andromeda. The database was offered by researchers.

### 2.16 Statistics

All experiments were performed at least in triplicate unless otherwise specified. Data are represented as mean ± standard deviation (SD). P values were calculated using the unpaired Student’s *t* test or Mann–Whitney *U* test, depending on the normality and homoscedasticity of the data. Statistical significance was defined as follows: n.s., P > 0.05; ∗, P < 0.05; ∗∗, P < 0.01; ∗∗∗, P < 0.001; ∗∗∗∗, P < 0.0001. Graphs were generated using GraphPad Prism 9, with bar charts displaying individual data points together with mean ± SD.

## 3 Results

### 3.1 *In vivo* selection of highly bone-metastatic cell line

**Fig. 1:**
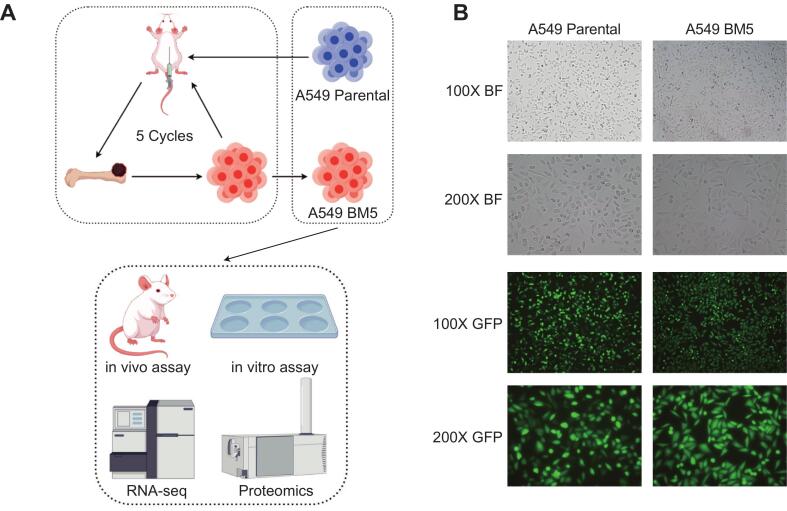
The *in vivo* selection of a highly bone-metastatic A549 subline. (A) Schematic diagram illustrating the establishment and characterization of the A549-BM5 subline. (B) Morphological characteristics of A549-Parental and A549-BM5 cells observed under bright-field microscopy and fluorescence microscopy (GFP channel) at 100× and 200× magnifications.

To establish a highly bone-metastatic subline derived from the lung adenocarcinoma A549 cell line, we utilized an *in vivo* selection strategy based on left ventricle injection (Fig. 1A). A549 cells labeled with GFP and luciferase were injected into the left ventricles of NOD/SCID mice to establish a lung cancer bone metastasis mouse model. In-vivo bioluminescent imaging (BLI) was immediately used to confirm successful injections, and BLI was then continuously utilized to monitor the progression of bone metastatic lesions. Approximately one month after injection, detectable bone metastatic lesions had emerged. At this point, the mice were humanely euthanized, and the bone metastatic tissues were collected, minced, and subjected to primary cell culture for subsequent in-vivo passaging. This sequence of left ventricle injection, primary culture, and reinjection was repeated for five consecutive cycles. During these cycles, both the incidence and the onset time of bone metastases significantly increased, thus validating the efficacy of this selection strategy. The cells obtained after five rounds of in-vivo selection were designated as A549-BM5. Fluorescence microscopy verified that all cells expressed GFP, demonstrating the successful derivation of a highly purified population of A549-derived cells from the xenografts (Fig. 1B). Bright-field microscopy showed that, in contrast to the typical adherent epithelial-like morphology of parental A549 cells, A549-BM5 cells adopted a more elongated, spindle-shaped appearance (Fig. 1B).

### 3.2 The *in vitro* phenotype of the A549-BM5 cell line

**Fig. 2:**
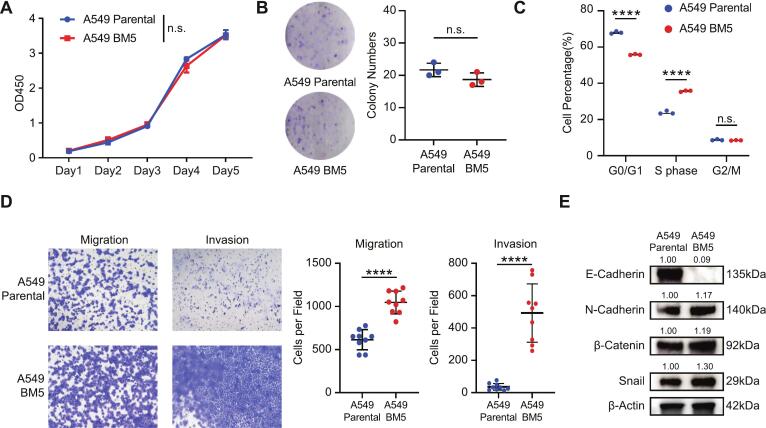
The *in vitro* functions and epithelial-mesenchymal status of A549-BM5 cell subline (A) Cell counting kit-8 assay: *in vitro* proliferation analysis of A549-Parental and A549-BM5 cells (n = 3). (B) Colony formation ability of A549-Parental and A549-BM5 cells (*n* = 3). (C) Flow cytometry was used to detect changes in the DNA content distribution in the A549-Parental and A549-BM5 cells (n = 3). (D) Transwell assay assessing the migration and invasion abilities of A549-Parental and A549-BM5 (n = 9). (E) Western blot analysis of E-Cadherin, N-Cadherin, β-Catenin and Snail in A549-Parental and A549-BM5 cells Data are represented as mean ± SD. *P* values were calculated using the unpaired Student’s *t* test or Mann-Whitney *U* test (Depending on the normality and homoscedasticity of the data). n.s., P > 0.05, ∗, *P* < 0.05; ∗∗, *P* < 0.01, ∗∗∗, *P* < 0.001, ∗∗∗∗, *P* < 0.0001.

To comprehensively clarify the *in vitro* characteristics of the A549-BM5 cell line, we conducted a detailed comparison between it and its parental cells with respect to proliferation, migration, and epithelial-mesenchymal status. Cell Counting Kit-8 (CCK-8) assays indicated that the A549-BM5 cell line did not show a proliferative advantage over the parental A549 cells *in vitro* (Fig. 2A). Additionally, the colony formation ability of the A549-BM5 cells was comparable to that of the parental cells (Fig. 2B). Cell cycle analysis revealed a reduction in the proportion of cells in the G0/G1 phase and an elevation in the S-phase population, while the proportion of cells in the G2/M phase remained largely unchanged when compared to the parental cells (Fig. 2C). Transwell assays clearly demonstrated that the A549-BM5 cells exhibited significantly enhanced migration and invasion capabilities compared to their parental counterparts (Fig. 2D). Western blot analysis showed a clear reduction of E-cadherin in A549-BM5 compared with parental A549 cells. Minor changes were also observed in N-cadherin, β-catenin, and Snail (Fig. 2E). Notably, we observed that E-cadherin is significantly downregulated, while N-cadherin, β-catenin, and Snail are upregulated. This pattern suggests the cells are undergoing epithelial-mesenchymal transition (EMT), becoming more migratory and invasive.

### 3.3 Interaction capacity of A549-BM5 cells with bone microenvironment cells

Tumor progression during bone metastasis relies on intricate interactions with a variety of cells in the bone microenvironment [8]. To investigate the interaction capacity of A549-BM5 cells with these cellular constituents, we examined their impacts on osteoclasts, osteoblasts, and their progenitor cells.

**Fig. 3:**
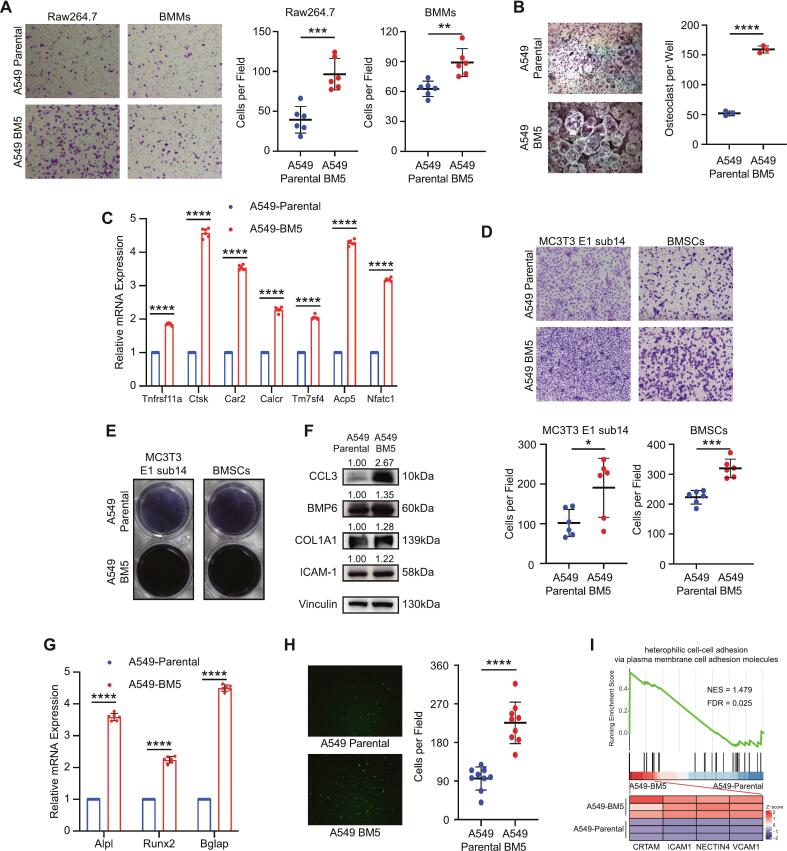
The capacity of A549-BM5 cells to interact with bone microenvironment cells. (A) Transwell assays showing the recruitment capacity of A549-Parental and A549-BM5 cells toward osteoclast precursors. Both RAW264.7 cells and bone marrow–derived macrophages (BMMs) were used as osteoclast precursor models. Quantification indicates significantly stronger recruitment in the A549-BM5 group (n = 6). (B) Osteoclast differentiation assay using BMMs. After 14 days of induction with conditioned medium (CM) from A549-Parental or A549-BM5 cells, TRAP staining and per-well quantification confirmed markedly enhanced osteoclast formation in the A549-BM5 group (n = 3). (C) RT-qPCR analysis of osteoclast differentiation markers (Tnfrsf11a, Ctsk, Car2, Calcr, Tm7sf4, Acp5, and Nfatc1) in BMMs cultured for 14 days with conditioned medium (CM) from A549-Parental or A549-BM5 cells. Gene expression was normalized to β-actin and calculated using the 2^−ΔΔCt^ method (n = 6). (D) Transwell assays showing recruitment capacity of A549-Parental and A549-BM5 cells toward osteoblast progenitors (MC3T3-E1 subclone 14 and BMSCs). Quantification demonstrates enhanced recruitment by A549-BM5 cells (n = 6). (E) Osteoblast differentiation assay. MC3T3-E1 subclone 14 cells and BMSCs were induced for 14 days with osteogenic medium containing CM from A549-Parental or A549-BM5 cells. ALP staining demonstrated stronger osteogenic differentiation in the A549-BM5 group (n = 3). (F) Conditioned media (CM) from A549-Parental and A549-BM5 cells were used to treat MC3T3 E1 sub14 cells for 14 Days, and the expression levels of CCL3, BMP6, COL1A1 and ICAM-1 were detected using Western blot analysis. (G) RT-qPCR analysis of osteoblast differentiation markers (Alpl, Runx2, and Bglap) in MC3T3-E1 subclone 14 cells cultured for 14 days with conditioned medium (CM) from A549-Parental or A549-BM5 cells. Gene expression was normalized to β-actin and calculated using the 2^−ΔΔCt^ method (n = 6). (H) Adhesion assay of GFP-labeled A549-Parental and A549-BM5 cells to MC3T3-E1 subclone 14 cells. Quantification showed that A549-BM5 cells exhibited significantly greater adhesion than parental cells (n = 9). (I) GSEA analysis shows that the GO:0007155 gene set (heterophilic cell–cell adhesion via plasma membrane cell adhesion molecules) is enriched in A549-BM5 cells compared with the A549-Parental group. The heatmap below displays the expression of leading-edge genes in the corresponding samples.Data are represented as mean ± SD. *P* values were calculated using the unpaired Student’s *t* test or Mann-Whitney *U* test (Depending on the normality and homoscedasticity of the data). n.s., P > 0.05, ∗, *P* < 0.05; ∗∗, *P* < 0.01, ∗∗∗, *P* < 0.001, ∗∗∗∗, *P* < 0.0001.

Using Transwell assays, we found that A549-BM5 cells exhibited a significantly enhanced ability to recruit osteoclast precursors compared with parental cells. Both RAW264.7 cells and bone marrow–derived macrophages (BMMs) were used as osteoclast precursor models, and in both conditions A549-BM5 cells demonstrated stronger recruitment (Fig. 3A). For osteoclast differentiation experiments, we specifically used BMMs isolated from C57BL/6J mice. After 14 days of culture under osteoclastogenic induction, A549-BM5 CM significantly promoted osteoclast formation, as confirmed by TRAP staining and per-well quantification (Fig. 3B). At the transcriptional level, RT-qPCR further revealed significant upregulation of osteoclast differentiation markers including Tnfrsf11a, Ctsk, Car2, Calcr, Tm7sf4, Acp5, and Nfatc1 in the A549-BM5 group (Fig. 3C).

To investigate osteoblast-related interactions, we employed MC3T3-E1 subclone 14 cells and primary BMSCs. Transwell assays showed that A549-BM5 cells significantly enhanced recruitment of osteoblast progenitors (Fig. 3D). Under osteogenic induction with CM for 14 days, osteoblast differentiation was markedly increased in the A549-BM5 group, as demonstrated by ALP staining (Fig. 3E).

We next evaluated the expression of potential mediators in MC3T3-E1 subclone 14 cells treated with conditioned medium (CM) for 14 Days by Western blot. A549-BM5 CM induced a clear increase in CCL3, with BMP6, COL1A1 and ICAM-1 showing a tendency to be higher (Fig. 3F). At the transcriptional level, RT-qPCR analysis of MC3T3-E1 subclone 14 cells cultured for 14 days under CM treatment further confirmed upregulation of osteoblast differentiation markers Alpl, Runx2, and Bglap in the A549-BM5 group (Fig. 3G).

Finally, adhesion assays showed that A549-BM5 cells displayed superior attachment to MC3T3-E1 cells compared with parental A549 (Fig. 3H). Consistently, GSEA revealed enrichment of the GO:0007155 gene set (heterophilic cell–cell adhesion via plasma membrane cell adhesion molecules) in A549-BM5 cells compared with the A549-Parental group, and the accompanying heatmap shows the expression of the leading-edge genes, including VCAM1, ICAM1, NECTIN4, and CRTAM, in the corresponding samples (Fig. 3I).

### 3.4 A549-BM5 cells exhibit enhanced bone metastatic potential *in vivo*

**Fig. 4:**
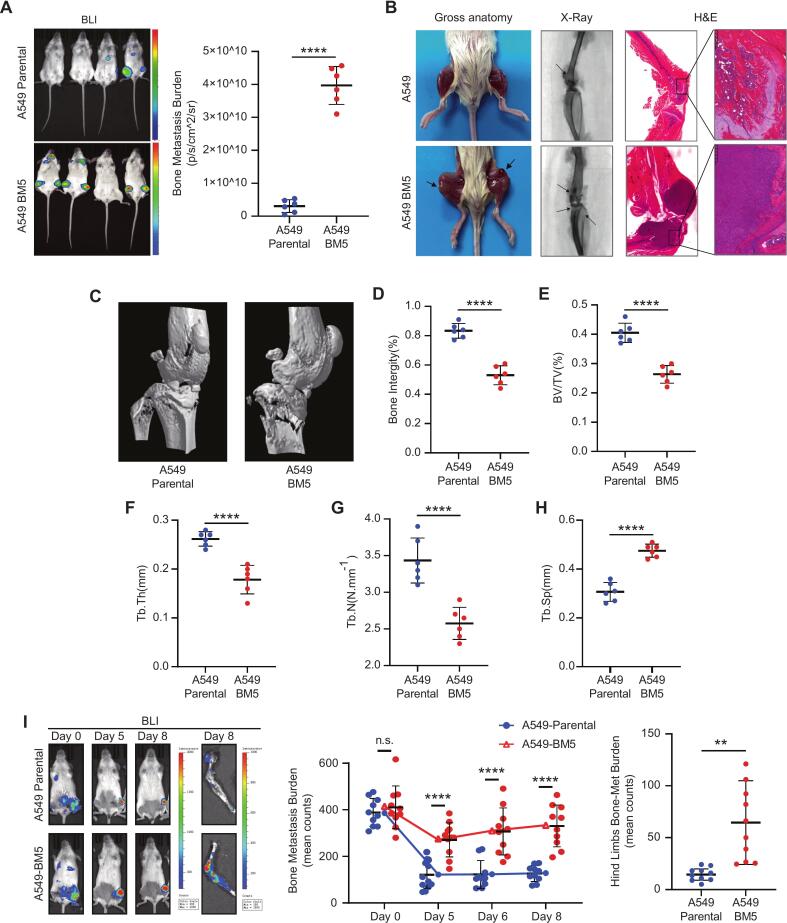
The *in vivo* metastatic capacity of A549-BM5 (A) An intra-cardiac injection was used to establish bone metastasis CDX models of A549-Parental and A549-BM5 cells. *In vivo* bioluminescent imaging (BLI) was employed to detect the bone metastatic burden (n = 6).(B) Representative gross anatomy, X-ray imaging and H&E staining of bone lesions from A549-Parental and A549-BM5 groups. Extensive osteolytic destruction was observed in A549-BM5–bearing mice. (C) Representative three-dimensional micro-CT images from A549-Parental and A549-BM5 groups. (D) Quantitative analysis of bone integrity, showing significantly reduced integrity in the A549-BM5 group compared with A549-Parental (n = 6). (E) Quantitative analysis of bone volume fraction (BV/TV), revealing markedly decreased bone mass in the A549-BM5 group (n = 6). (F) Quantitative analysis of trabecular thickness (Tb.Th), showing thinner trabeculae in A549-BM5 mice (n = 6). (G) Quantitative analysis of trabecular number (Tb.N), indicating fewer trabeculae in the A549-BM5 group (n = 6). (H) Quantitative analysis of trabecular spacing (Tb.Sp), showing significantly increased spacing in the A549-BM5 group (n = 6). (H) Intra-iliac artery injection model of hindlimb bone metastasis. Representative longitudinal BLI monitoring shows significantly higher tumor burden in the A549-BM5 group compared to A549-Parental. Quantification of bone metastasis burden over time (left panel) and ex vivo BLI of hindlimb bone tissues at day 8 (right panel) are shown (n = 10). Data are represented as mean ± SD. *P* values were calculated using the unpaired Student’s *t* test or Mann-Whitney *U* test (Depending on the normality and homoscedasticity of the data). n.s., P > 0.05, ∗, *P* < 0.05; ∗∗, *P* < 0.01, ∗∗∗, *P* < 0.001, ∗∗∗∗, *P* < 0.0001.

To investigate the bone metastatic potential of A549-BM5 cells *in vivo*, we established mouse models using both left ventricular and intra-iliac artery injections. Following left ventricular injection, the progression of skeletal metastasis was dynamically monitored by bioluminescent imaging (BLI). All mice injected with A549-BM5 cells developed substantial bone metastatic lesions within 14 days, whereas parental A549 cells showed only limited colonization. At the endpoint, A549-BM5–bearing mice exhibited a significantly elevated bone metastatic burden, which was further confirmed by gross anatomy, X-ray imaging, micro-CT reconstruction, and histological examination (Fig. 4A–C). Quantitative micro-CT analysis revealed pronounced osteolytic bone destruction in A549-BM5 mice, including reduced bone integrity, decreased bone volume fraction (BV/TV), trabecular thickness (Tb.Th), and trabecular number (Tb.N), along with increased trabecular spacing (Tb.Sp) (Fig. 4D–H).

To further assess whether A549-BM5 cells could effectively mimic early biological events of bone colonization, we established a unilateral hindlimb metastasis model via intra-iliac artery injection. Notably, differences in metastatic burden became apparent as early as day 5–7, with BLI and ex vivo analyses showing markedly higher tumor load in A549-BM5 compared to parental A549 controls (Fig. 4I).

### 3.5 Transcriptomic and proteomic analyses identify potential metastatic biomarkers

**Fig. 5:**
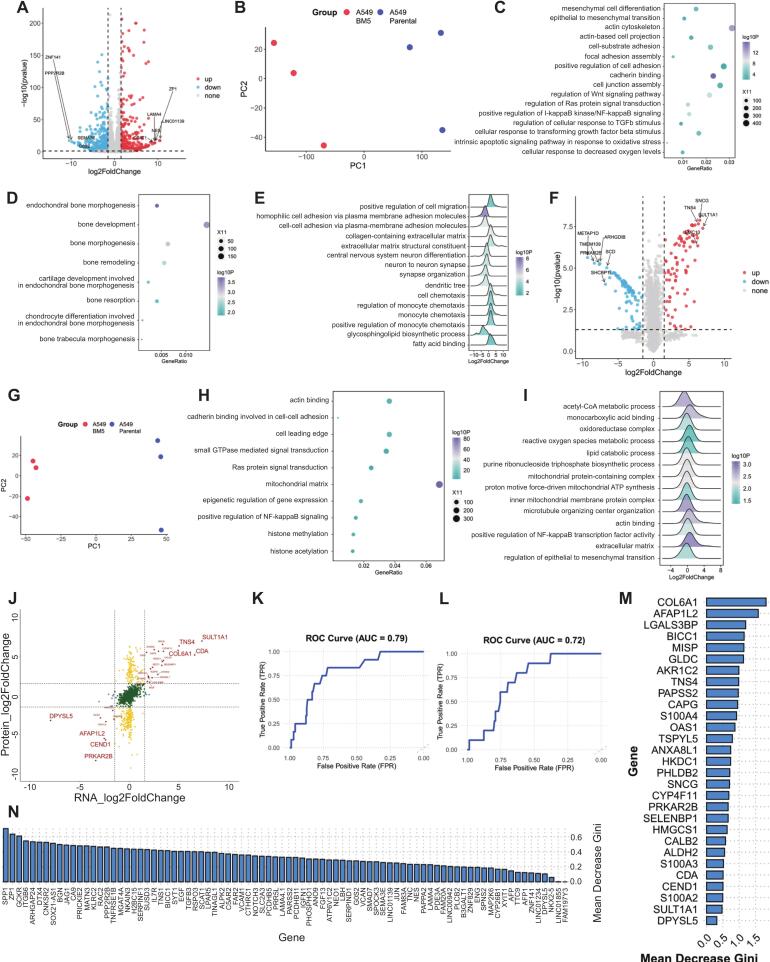
Analysis of the transcriptomic and proteomic characteristics of A549-BM5. (A) The volcano plot generated from transcriptome analyses of RNA sample from A549-Parental and A549-BM5 (n = 3). (B) Principal component analysis (PCA) of RNA sequencing data from A549-Parental and A549-BM5 cells. (C) The bubble chart illustrates the Gene Ontology (GO) enrichment analysis of differentially expressed genes (DEGs) between A549-Parental and A549-BM5 cells. (D) The bubble chart illustrates the enrichment of bone-related gene sets in the Gene Ontology (GO) enrichment analysis of differentially expressed genes (DEGs) between A549-Parental and A549-BM5 cells. (E) Gene Set Enrichment Analysis (GSEA) using the GO database in the transcriptomic analysis. (F) The volcano plot generated from proteomic analyses of A549-Parental and A549-BM5 (n = 3). (G) Principal component analysis (PCA) of proteomic data from A549-Parental and A549-BM5 cells. (H) The bubble chart illustrates the Gene Ontology (GO) enrichment analysis of differentially expressed proteins between A549-Parental and A549-BM5 cells; (I) Gene Set Enrichment Analysis (GSEA) using the GO database in the proteomic analysis. (J) The Nine-square Grid displays genes that have common changing trends between proteomic and transcriptomic analyses. (K–L) Using the LUAD cohort from TCGA, two random forest models were constructed to evaluate the contribution of genes to metastasis (M1). The ROC curves respectively display the classification performance of models established using commonly altered genes (K) and characteristic genes selected from the transcriptomic data (L). (M) Mean Decrease Gini of genes exhibiting concurrent changes in transcriptomic and proteomic profiles. (N) Mean Decrease Gini of characteristic genes in transcriptomic profiles.

We conducted a comprehensive characterization of A549-BM5 cells at the transcriptomic and proteomic levels. mRNA sequencing was performed on A549-BM5 cells and their parental counterparts. Using a filtering threshold of a fold change greater than 2 and an adjusted p-value less than 0.05, we identified 1,371 differentially expressed genes (DEGs), including 589 upregulated and 782 downregulated genes (Fig. 5A). Principal component analysis (PCA) revealed a clear separation between A549-BM5 cells and their parental counterparts (Fig. 5B). Gene Ontology (GO) enrichment analysis identified multiple gene sets associated with epithelial-mesenchymal transition (EMT), cell–cell adhesion, the WNT signaling pathway, the NF-κB signaling pathway, and the transforming growth factor (TGF) pathway (Fig. 5C), all of which known to play key roles in bone metastasis. Additionally, bone-related gene sets, including bone tissue morphogenesis and development, were significantly enriched (Fig. 5D), which may indicate that A549-BM5 cells acquire osteogenic-like transcriptional features. Gene Set Enrichment Analysis (GSEA) further revealed enrichment of gene sets related to cell migration, adhesion, neuron differentiation, and cytokine signaling (Fig. 5E).

To complement the transcriptomic data, we performed quantitative proteomics analysis on A549-BM5 and parental cells. Based on filtering criteria of a fold change greater than 1.5 and an adjusted p-value less than 0.05, we identified 144 upregulated proteins and 193 downregulated proteins (Fig. 5F). PCA of the proteomic data also demonstrated a clear separation between A549-BM5 cells and their parental counterparts (Fig. 5G). GO enrichment analysis and GSEA of the proteomic data revealed significant enrichment of pathways involved in cell migration, adhesion, Ras signaling, mitochondrial function, histone modification, EMT, and various metabolism − related pathways (Fig. 5H-I). Integrated analysis of transcriptomic and proteomic data identified 21 genes that were concurrently upregulated and 8 genes that were concurrently downregulated at both levels (Fig. 5H–J).

To explore the potential roles of these key genes in the distant metastasis of lung adenocarcinoma (LUAD) patients, we retrieved LUAD patient data from the TCGA database. Patients were stratified into M0 (no distant metastasis) and M1 (distant metastasis) groups according to their metastatic status. By utilizing a random forest model, we assessed the contribution of target genes to the metastatic status (Fig. 5K–L). Potential biomarkers for LUAD metastasis were identified based on their Mean Decrease Gini scores obtained from the random forest model (Fig. 5M–N).

## 4 Discussion

Recent single-cell analyses have revealed substantial intra-line heterogeneity in gene expression and metastatic potential within cancer cell lines, supporting the rationale for selecting organ-tropic sublines *via in vivo* selection [[23], [24], [25]]. Given the unique physicochemical properties and cytokine milieu of the bone and bone marrow microenvironment—characterized by hypoxia [26], high extracellular calcium levels [27], abundant matrix proteins such as collagen [28] and osteopontin [29], and soluble factors like TGF-β, CXCL12, and RANKL [8]—*in vivo* circulation selection enables effective isolation and enrichment of tumor subpopulations with a propensity for bone metastasis. The A549 cell line is a widely used model for studying lung adenocarcinoma [30]. However, our previous study revealed that although A549 cells possess the capacity to metastasize to bone after intracardiac injection, their metastatic efficiency is limited, with low and variable engraftment and a dispersed pattern of skeletal colonization. These features indicate the absence of strong bone-tropic preference and reduce their suitability for generating consistent and biologically relevant bone metastasis models [31]. Such characteristics make A549 suboptimal for quantitative studies specifically focused on bone metastasis.

In the following analysis, we examined the key features of currently available lung cancer cell models with high bone-metastatic potential. Hung *et al.* obtained results similar to our previous findings when establishing a bone metastasis model by injecting A549 cells *via* the left ventricle [32]. While bone lesions were observed, notable metastatic spread also occurred in other organs, including the lungs and abdominal viscera, potentially complicating the interpretation of bone-specific metastatic mechanisms. Tan *et al.* established a bone metastasis model *via* intra-tibial injection of A549 cells [33]. While this approach efficiently induces bone lesions, it bypasses key early steps of the metastatic cascade, limiting its relevance for mechanistic studies. Moreover, the invasive procedure introduces tissue injury and allows cancer cells to enter circulation, increasing the risk of systemic spread. Recent findings also suggest that injury-induced bone remodeling involving NG2^+^ stromal cells may artificially promote tumor colonization, further compromising model specificity [34]. This limitation was partially addressed by Cai et al*.*, who established the A549L6 subline through *in vivo* selection, which preferentially metastasizes to the spine. While A549L6 partially mimics the clinical pattern of lung cancer bone metastasis—typically initiating in the axial skeleton—it is less amenable to *in vivo* monitoring and is suboptimal for studying metastases to appendicular bones. In contrast, our A549-BM5 subline exhibits a broader and more consistent bone-tropic pattern, including frequent involvement of limb bones, thereby offering a more versatile and observable model for investigating skeletal colonization and therapeutic interventions [35]. However, given the unique biological features of vertebral bone—such as its distinct skeletal stem cell populations and specialized metastatic niche—this model may be more appropriate for investigating spine-specific metastasis rather than broadly representative skeletal dissemination [36]. Collectively, these limitations underscore the urgent need for a lung adenocarcinoma model that not only exhibits stable and reproducible bone tropism but also accurately reflects the general biological characteristics of skeletal metastasis—criteria that A549-BM5 is well positioned to fulfill. Given the clinical characteristics of bone metastasis, identifying biomarkers and therapeutic targets associated with early biological events of skeletal colonization holds significant research value. In this study, we evaluated the performance of the A549-BM5 subline in combination with intra-iliac artery injection to model this process. Remarkably, A549-BM5 exhibited a clear proliferative advantage as early as five days post-injection, suggesting that it effectively recapitulates several key niches involved in the early stages of bone metastasis, including the perivascular niche and the osteogenic niche.

Consistent with the notion that organ-specific microenvironments can drive phenotypic divergence even among genetically similar cancer cells [37,38], A549-BM5 exhibited not only stable proliferation *in vitro* but also acquired distinct metastatic traits—including reduced cell adhesion, elevated EMT marker expression, and significantly enhanced migratory and invasive capacities. These features, coupled with its robust and early skeletal colonization *in vivo*, suggest that repeated exposure to the bone microenvironment exerted strong selective pressure, enriching for a tumor subpopulation with specialized bone-homing potential. Mechanistically, several components of the bone niche may have contributed to this phenotypic evolution. Hypoxic conditions, characteristic of specific niches within the bone marrow microenvironment [39,40], are known to activate HIF-1α signaling, which in turn promotes EMT and enhances metastatic capacity [41]. TGF-β, abundantly released during osteolytic bone remodeling, has also been shown to induce mesenchymal transition and support metastatic seeding [9]. Additionally, extracellular matrix proteins such as collagen and osteopontin, along with mechanical cues like increased matrix stiffness, can activate integrin and focal adhesion kinase (FAK) signaling pathways to further promote tumor cell motility and survival [42,43]. Moreover, accumulating evidence indicates that even subtle environmental cues can induce long-lasting transcriptional and epigenetic reprogramming in tumor cells during *in vivo* selection [44,45]. Therefore, the stable acquisition of bone-adaptive traits in A549-BM5 may reflect not only selective outgrowth of pre-existing subclones, but also microenvironment-driven plasticity that enhances tumor cell fitness in the skeletal niche.

Importantly, the clinical relevance of the A549-BM5 model was further substantiated by transcriptomic and proteomic analyses, which not only revealed classical molecular hallmarks of bone metastasis but also aligned closely with clinical observations. Previous studies in prostate cancer have shown that once tumor cells colonize the bone marrow microenvironment, they acquire osteomimicry features—such as the expression of alkaline phosphatase, osteocalcin, osteopontin, and bone morphogenetic proteins (BMPs)—to support their survival and progression within bone tissue [46,47]. Interestingly, A549-BM5 displayed a similar trend, with a transcriptomic profile resembling that of osteogenic cells and robust activation of the WNT signaling pathway—a critical regulator of both osteogenesis and bone metastasis—further reinforcing its bone-adaptive phenotype [46]. In addition, A549-BM5 showed marked enrichment of neuron-associated gene sets, suggesting a possible contribution of neurobiological processes to its metastatic behavior. Although the role of neural regulation in lung cancer bone metastasis has been relatively underexplored, emerging evidence is beginning to highlight its importance. For instance, one study revealed that sensory nerves expressing calcitonin gene–related peptide (CGRP) are enriched at bone metastatic sites and promote tumor progression *via* the CGRP/CRLR/p38/HSP27 signaling axis [48]. These findings provide compelling support for leveraging A549-BM5 as a preclinical model to explore mechanisms underlying bone metastasis.

Taken together, A549-BM5 offers a stable, bone-tropic, and clinically relevant model for lung cancer bone metastasis, characterized by distinct transcriptomic and proteomic alterations compared to the parental A549 line and enhanced interactions with key components of the bone microenvironment. These features make it well-suited for mechanistic studies and therapeutic exploration targeting the skeletal niche.

### 4.1 Limitations of the study

The immune system plays an important role in the process of bone metastasis [49,50]. Due to the limitations of Lewis lung cancer cells, we chose A549 cells to establish a highly bone-metastatic subline, which limits the possibilities for immune-related studies. Moreover, the *in vivo* selection strategy based on intracardiac injection bypasses the early steps of the metastasis cascade, such as pre-metastatic alterations in bone [51], the rise of bone-tropic metastatic seeds in primary tumors [52], and the perivascular niche and metastasis dormancy [53].

## Author contributions

Y.J.X., Y.H.Q, W.J.C., X.K., X.L.L, L.L., J.L. Y.C. and L.S. conducted the experiments; H.Y.P. and M.X.Y. designed the experiments, Y.J.X. wrote the paper.

## 6 Materials availability

This study did not generate new unique reagents.

## 7 Data and code availability

There is no original code in this study.

Any additional information required to reanalyze the data reported in this paper is available from the lead contact upon request.

## CRediT authorship contribution statement

**Yujian Xu:** Writing – original draft, Visualization, Methodology, Investigation, Data curation, Conceptualization. **Yahan Qin:** Software, Methodology, Investigation. **Wenjun Chai:** Resources, Methodology, Formal analysis. **Ke Xue:** Software, Data curation. **Xiaoli Liu:** Supervision. **Jing Li:** Supervision. **Yue Cao:** Validation. **Lei Sun:** Supervision, Methodology. **Hongyu Pan:** Writing – review & editing, Supervision, Conceptualization. **Mingxia Yan:** Writing – review & editing, Supervision, Methodology, Funding acquisition, Conceptualization.

## Declaration of competing interest

The authors declare that they have no known competing financial interests or personal relationships that could have appeared to influence the work reported in this paper.
